# Preparation and Coagulation Performance of Carboxypropylated and Carboxypentylated Lignosulfonates for Dye Removal

**DOI:** 10.3390/biom9080383

**Published:** 2019-08-20

**Authors:** Khatereh Bahrpaima, Pedram Fatehi

**Affiliations:** 1 Department of Chemical Engineering, Lakehead University, Thunder Bay, ON P7B 5E1, Canada; 2 Department of Chemistry, Firoozabad Branch, Islamic Azad University, Firoozabad 74719-13113, Iran

**Keywords:** polymer material, biorefining, coagulation, carboxyalkylation, NMR, lignin

## Abstract

In this work, 1-carboxypropyled (1-CPRLS) and 5-carboxypentyled lignosulfonates (5-CPELS) were synthesized using 2-chlorobutanoic acid and 6-chlorohexanoic acid as carboxylate group donors via S_N_1 and S_N_2 mechanisms, respectively. 1-Carboxypropyl and 5-carboxypentyl lignosulfonates with the charge densities of −3.45 and −2.94 meq g^−1^ and molecular weights of 87,900 and 42,400 g·mol^−1^ were produced, respectively, under mild conditions. The carboxylate content and degree of substitution (DS) of the 1-CPRLS product were 2.37 mmol·g^−1^ and 0.70 mol·mol^−1^, while those of 5-CPELS products were 2.13 mmol·g^−1^ and 0.66 mol·mol^−1^, respectively. The grafting of carboxypropyl and carboxypentyl groups to lignosulfonate was confirmed by Fourier transform infrared (FT-IR) and nuclear magnetic resonance (^1^H-NMR and ^13^C-NMR) spectroscopies. In addition, 1-CPRLS and 5-CPELS were applied as coagulants for removing ethyl violet (EV) dye from a simulated solution, and their performance was related to their charge densities and molecular weights. Furthermore, fundamental discussion is provided on the advantages of (1) producing 1-CPRLS and (2) the superior properties and performance of 1-CPRLS to carboxyethylated lignosulfonate.

## 1. Introduction

The importance and logistical necessity of providing an alternative to the expensive fossil-based materials has been comprehensively discussed [1,2,3,4,5]. As an example, fossil-based fuels can be replaced by non-food waste biomass generated in the pulping industry [1,2,3,4,5].

Lignin is a lignocellulosic biomass-derived multifunctional molecule that is considered a useful by-product with high valorization potential for producing value-added products [6,7]. In this regard, lignosulfonate as a by-product of spent sulfite liquor [8,9,10,11] can be recovered and valorized into high-grade commercial products using novel derivatization and green engineering approaches [12,13,14,15,16,17,18,19,20]. Lignosulfonate derivatives were reported to be used as emulsifiers/emulsion stabilizers, surfactants for pesticides, oil recovery and drilling muds, concrete cure retarders and plasticizers, and binders for foundry and wood adhesives [21]. However, the use of lignosulfonate for dye removal may expand its application to wastewater treatment in the textile industry.

Currently, conventional biological treatment processes are not very effective in treating dye wastewaters, and the research community is exploring opportunities for identifying an alternative process for removing dyes from wastes [22,23]. Today, the effluents of textile, painting, paper and pulp, leather, and cosmetic industries contain significant amounts of natural and synthetic dyes. The high solubility of these toxic dyes in water and the environmental pollution caused by them makes these dyes harmful to the health of aquatic life, humans, and other mammals [24,25]. Therefore, the application of economical and eco-friendly separation techniques—specifically, coagulation/flocculation for water recovery—is essential to a pollution-free environment [26,27,28,29]. In this context, one of the candidate materials is lignosulfonate, which can be properly chemically functionalized and used as an anionic polymeric coagulant for dye removal.

Different reaction pathways have been used in the literature for functionalizing lignosulfonate. Examples of effective modification and functionalization techniques are desulfonation graft polymerization, sulfomethylation, esterification, nitration, oxidation, hydroxyalkylation, phenolation, and sulfonation [21,30]. Very recently, various carboxyalkylation derivatizations of biomass were also carried out [31,32,33,34,35,36,37,38,39,40,41,42,43,44,45,46,47,48,49,50,51,52,53,54,55,56].

Carboxyalkylation provides high anionic charges to lignosulfonate that facilitate its interaction with dyes. Carboxyalklylation provides a carboxylic acid group to lignosulfonate, improving its charge interaction in colloidal systems. The chain length of the grafting group and the reaction environment can be altered by following different carboxyalkylation routes in order to produce carboxyalkylated lignosulfonate with improved charge density and molecular weight. In our previous work, we studied the effects of the carboxyethylation of lignosulfonate on the physicochemical properties of the product (1-CELS) [20]. Despite its advantages, more research is still needed to produce a more efficient functional lignosulfonate (LS). Following this pathway, this study focused on the preparation of modified LS containing longer hydrocarbon chains with a carboxylate group (i.e., 1-carboxypropyl and 5-carboxypentyl) to achieve carboxyalkylated lignosulfonate with larger molecular weight compared with carboxyethylated lignosulfonate (CELS). It has been reported that the flocculation process can be promoted by the molecular weight of polymers, as large polymers might bridge particles in solution [26,28].

In this work, various advanced tools, such as ^1^H-NMR, ^13^C-NMR, GPC, FT-IR, potentiometric titration, charge density, and elemental analyses were used for product characterization. The present study also emphasizes the optimization of carboxypropylation and carboxypentylation reactions using the Taguchi method [31,57,58,59,60]. As 1-CPRLS and 5-CPELS are produced by substituting lignosulfonate’s -OH groups and lignosulfonate has different aliphatic and aromatic-OH groups, this study aims at identifying the fate of these groups in the graft modification process.

Carboxyalkylated lignosulfonates may have great potential to serve as efficient eco-friendly coagulants for cationic dyes [22]. Ethyl violet, a synthetic triphenylmethane dye, is extensively used in various industrial dyeing processes, and can be released to wastewater [25,61]. Therefore, the removal of this dye from wastewater is crucial. In the current research, 1-CPRLS and 5-CPELS, as new eco-friendly coagulants, were used for removing cationic ethyl violet (EV) from a simulated solution.

## 2. Materials and Methods

### 2.1. Chemicals

Lignosulfonic acid sodium salt (97.0%), sodium hydroxide (99.0%), potassium hydroxide (8.0 N), D_2_O isotopic purity (99.8%), hydrochloric acid (37.0%), 2-chlorobutanoic acid (99.0%), 6-chlorohexanoic acid (99.0%), 4-hydroxybenzoic acid (98.0%), ethanol (99.1%), isopropyl alcohol (99.8%), sodium nitrate (99.8%), dimethyl sulphate (98.0%), acetic anhydride (99.0%), polyethylene oxides (99.0%), 3-trimethylsilyl-(2,2,3,3-d4)-propionic acid sodium salt (TSP) (99.8%), polydiallyldimethylammonium chloride (PDADMAC) (20 wt.%), and chromium (III) acetylacetonate Cr (acac)_2_ (99.99%), all of analytical grades, were purchased from Sigma-Aldrich, Oakville, ON or BOC Sciences, Shirley, NY, USA. Dialysis tube (Mw cut-off 10,000 g/mol) was obtained by Thermo Fisher Scientific Inc., Ottawa, ON, Canada.

### 2.2. Synthesis of 1-Carboxypropyl Lignosulfonate and 5-Carboxypentyl Lignosulfonate

Following the procedure described for the carboxyethylation of lignosulfonate [20], 1.5 g of lignosulfonate was suspended in a mixture of isopropyl alcohol (45 mL) and 30 wt.% NaOH (5, 8, and 11 mL) at 25 °C for 30 min. The activated lignosulfonate was then reacted with chloro carboxylic acid (ClCBA_i_/LS (1.0, 1.5, and 2.0 mol·mol^−1^), ClCBA_i_ including 2-chlorobutanoic acid or 6-chlorohexanoic acid) under altered times and temperatures in 250 mL three-neck round-bottom glass flasks under constant stirring at 300 rpm [62]. The product was extensively washed with ethanol/water (40:10, *v*/*v*) and recovered by centrifugation (3500 rpm, 10 min). After three cycles of washing/centrifugation, the precipitate was dissolved in deionized water (50 mL), then purified using dialysis tubes in deionized water for two days, and finally dried in an oven at 105 °C overnight. Different 1-carboxypropyl lignosulfonate and 5-carboxypentyl lignosulfonate samples were prepared by employing different reaction times (1.0, 1.5, and 2.0 h), temperatures (60, 70, and 80 °C), ClCBA_i_/LS ratios (1.0, 1.5, and 2.0 mol·mol^−1^), and water/isopropyl alcohol ratios (10%, 15% and 20%). It should be stated that 1-CPRMLS and 5-CPEMLS were produced following the same procedures of 1-CPRLS and 5-CPELS under the conditions of 1.0 ratio of ClCBA_i_/MLS by mol·mol^−1^, 80 °C, 2.0 h, and 15% water/isopropyl alcohol ratio.

### 2.3. Methylated Lignosulfonate

For this modification route, 2.0 g of LS was dissolved in 30 mL of 0.7 mol·L^−1^ NaOH solution in a 250 mL three-neck glass flask at room temperature by stirring at 200 rpm for 30 min. In a glass beaker, 2.5 mmol of dimethyl sulphate was added per each mmol of total phenolic hydroxyl groups of LS, and the solution was stirred at room temperature for 30 min. The mixture was then heated to 80 °C for an additional 2 h. During the reaction, the pH of the mixture was maintained at 11.0–11.5 by continuous addition of 0.7 mol·L^−1^ NaOH solution. After the reaction, the suspension was acidified to pH 2.5 by adding 2.0 mol·L^−1^ HCl solution, washed with excess deionized water, dialyzed for two days, and finally dried in a freeze-dryer. The final product was denoted as methylated LS (MLS) [20,63,64].

### 2.4. Taguchi Experimental Design

Taguchi’s orthogonal array (OA) was used to obtain the maximum charge density and molecular weight of 1-CPRLS and 5-CPELS under the optimized conditions [31,57,58,59,60]. In this study, a total of nine runs (L_9_) with three factors (each at four levels) were conducted based on an orthogonal design to investigate the effect of parameters on the carboxyalkylation reactions [20].

### 2.5. Functional Group Analysis

The aqueous potentiometric titration method [20,63] was used to evaluate the phenolic hydroxyl and carboxylate group contents of samples using an automatic potentiometer titrator (785 DMP Titrino, Metrohm, Switzerland). The degrees of substitution (DSs, mol·mol^−1^) of carboxypropyl and carboxypentyl groups were calculated using Equation (1) [20,31,65]:

(1) $$\mathrm{DS}=\frac{M\times A}{1-\mathrm{MC}\times A},$$

where A is the total carboxylate group content (mmol·g^−1^), M is the mass of the primary unit of lignosulfonate (0.223 g·mmol^−1^), and MC is the mass of sodium carboxypropyl (0.109 g·mmol^−1^) or carboxypentyl groups (0.137 g·mmol^−1^), respectively.

### 2.6. Charge Density Analysis

In preparing the samples for this analysis, LS, MLS, 1-CPRLS, 5-CPELS, 1-CPRMLS, and 5-CPEMLS were firstly dried in a 105 °C oven overnight to remove moisture. A 0.1 g sample of each lignosulfonate derivative was added to 10 mL of deionized water, then it was used for determining their charge density using a particle charge detector (Mutek, PCD 04, BTG Instruments, GmbH, Weßling, Germany) with a PDADMAC standard solution (0.006 M) [31]. The reported data in this paper are the average of three repetitions.

### 2.7. Molecular Weight Analysis

Molecular weight analysis was performed for LS, MLS, 1-CPRLS, 5-CPELS, 1-CPRMLS, and 5-CPEMLS samples using gel permeation chromatography, Malvern GPCmax VE2001 Module + Viscotek TDA305 with viscometer detectors. About 20–30 mg of the dried samples were dissolved in 10 mL of 0.1 mol·L^−1^ NaNO_3_ solution and filtered with a 0.2 μm nylon filter. The filtered solutions were used for molecular weight analysis [30]. PolyAnalytic PAA206 and PAA203 columns were used at the column temperature of 35 °C. A 0.1 mol·L^−1^ NaNO_3_ solution was used as solvent and eluent. The flow rate was set at 0.70 mL·min^−1^. Polyethylene oxides were used as standard [31,63].

### 2.8. Elemental Analysis

Elemental analysis of LS, MLS, 1-CPRLS, 5-CPELS, 1-CPRMLS, and 5-CPEMLS was assessed using an Elementar Vario EL Cube elemental analyzer by the combustion method [66]. In order to remove any moisture, all samples were firstly dried in a 105 °C oven overnight. A 0.002 g quantity of each sample was used for determining their carbon, hydrogen, nitrogen, and oxygen contents.

### 2.9. FT-IR Analysis

Approximately 0.05 g of oven-dried LS, MLS, 1-CPRLS, 5-CPELS, 1-CPRMLS, and 5-CPEMLS were used for their structural characterization using FT-IR spectroscopy (Bruker Tensor 37, ATR accessory Ettlingen, Germany). The spectra were recorded in transmittance mode in the range between 600 and 4000 cm^−1^ with a 4 cm^−1^ resolution, and 32 scans per sample were conducted [20].

### 2.10. ^1^H-NMR Analysis

The LS, MLS, 1-CPRLS, 5-CPELS, 1-CPRMLS, and 5-CPEMLS were characterized by an INOVA-500 MHz instrument (Varian, USA). All dried samples were dissolved in D_2_O or dry DMSO-d_6_. All signals were referenced to the internal standard, trimethylsilylpropanoic acid (TSP), at 0.00 ppm chemical shift [31]. For the analysis, 45–50 mg of LS, MLS, 1-CPRLS, 5-CPELS, 1-CPRMLS, and 5-CPEMLS were dissolved in 700 μL of D_2_O containing TSP (1 mg TSP/1 mL D_2_O) at 25 °C for 30 min and 150 rpm. Alternatively, 25 mg of LS, 1-CPRLS, and 5-CPELS were dissolved in 500 μL of DMSO-d_6_ containing 2 mg of TSP at 25 °C overnight at 150 rpm. The NMR spectra were recorded at room temperature after 16 scans. A 45° pulse flipping angle, a 4.6 μs pulse width, a 2.05 s acquisition time, and a relaxation delay time of 1.00 s were considered in this analysis.

### 2.11. ^13^C-NMR Analysis

All NMR spectra were recorded using an INOVA-500 MHz instrument (Varian, USA), operated at 50 °C utilizing a mixture of DMSO-d_6_ and D_2_O as the solvent. The chemical shifts were referred to the solvent signal of DMSO-d_6_ at 39.5 ppm. The major disadvantage of ^13^C-NMR spectroscopy is its inherent low sensitivity, which requires that a concentrated sample/solvent solution be made [67]. Therefore, in this study, for qualitative ^13^C NMR, 125 mg of the samples was dissolved in 500 μL of DMSO-d_6_ and 50 μL of D_2_O at 50 °C overnight at 100 rpm. Chromium (III) acetylacetonate (5 mg) was also added to the solution to provide complete relaxation of all nuclei. Line broadening of 1 Hz was applied to FIDs before Fourier transform [68]. To obtain a satisfactory signal-to-noise ratio, minimizing baseline and phasing distortions, the ^13^C NMR spectra of LS, 1-CPRLS, and 5-CPELS were recorded at 50 °C (in order to reduce the viscosity of the solution) with a pulse angle of 90 °C, a pulse delay of 2 s, an acquisition time of 1.1 s, and a transient number of about 30,000. The total experiment time was 28–30 h. Finally, the inverse-gated decoupling sequence was used, which involved turning off the proton decoupler during the recovery between pulses so that the NOE effect was avoided.

### 2.12. Dye Removal Analysis

At first, 100 mg·L^−1^ of EV dye solution was prepared by dissolving the dye in deionized distilled water, and the solution was shaken for 10 min. This dye solution was considered as a model wastewater sample of the textile industry. In this set of experiments, 1 g·L^−1^ samples of LS, 1-CELS, 1-CPRLS, and 5-CPELS solutions were made by mixing each of them with deionized distilled water at room temperature. The mixtures were added to 10 mL of a 100 mg·L^−1^ dye solution in centrifuge tubes. Then, the tubes were maintained in a water bath shaker at 30 °C and 150 rpm for 10 min. The tubes were then centrifuged at 3000 rpm for 10 min using a Sorvall ST 16 Laboratory Centrifuge. The filtrates were collected for analysis, while precipitates were discarded. The concentrations of dye in the mixtures before and after treatments were determined using a Genesys 10 s UV–Vis spectrophotometer at the wavelength of 595 nm for EV. The dye removal was calculated by the following equation:

(2) $$\mathrm{Dye}\;\mathrm{removal},\;\mathrm{wt}.\%=\frac{C_{0}-C}{C_{0}}\times 100,$$

where C_0_ (g·L^−1^) and C (g·L^−1^) are the concentrations of the dye solution before and after the LS, 1-CELS, 1-CPRLS, and 5-CPELS treatment, respectively.

## 3. Results and Discussion

### 3.1. Mechanism of 1-Carboxypropylation of Lignosulfonate

**Scheme 1:**
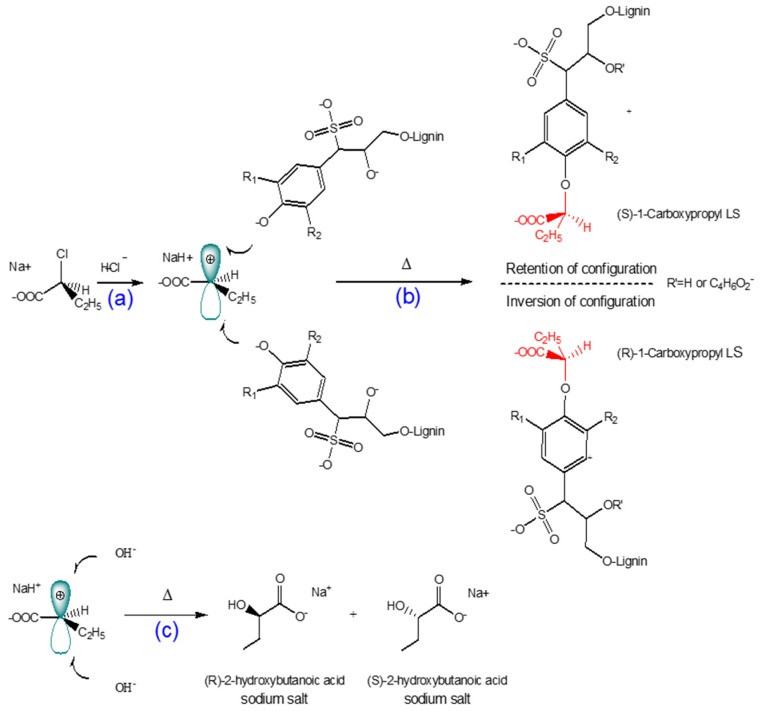
Proposed reaction mechanism for the carboxypropylation of lignosulfonate via an S_N_1 pathway: (**a**) formation of carbocation; (**b**) reaction of carbocated chemical with bulky nucleophile [66,69]; (**c**) undesired 2-hydroxybutanoic acid sodium product.

The reaction scheme of 1-carboxypropyled lignosulfonate is shown in Scheme 1. The carboxypropylation of lignosulfonate was performed using 2-chlorobutanoic acid as the carboxylate group donor in an alkali medium. Because 2-chlorobutanoic acid is a secondary halide, the reaction would take place via an S_N_1 mechanism in a water-isopropanol mixture as a polar protic solvent. Under alkali conditions, NaOH reacts with the hydroxyl group of lignosulfonate and generates a strong nucleophile. The dissociation of the carbon–halogen bond in 2-chlorobutanoic acid creates a planar carbocation (Scheme 1a). The alkoxide ion from the alkali lignosulfonate attacks the carbocation intermediate, resulting in the carboxypropylation reaction (Scheme 1b) [66,69]. The degree of carboxypropylation depends on the number of hydroxyl groups substituted by carboxypropyl groups. Furthermore, 2-hydroxybutanoic acid sodium salt can be generated as a by-product at high concentrations of 2-chlorobutanoic acid and NaOH (Scheme 1c) [20,66,69].

### 3.2. Mechanism of 5-Carboxypentylation of Lignosulfonate

**Scheme 2:**
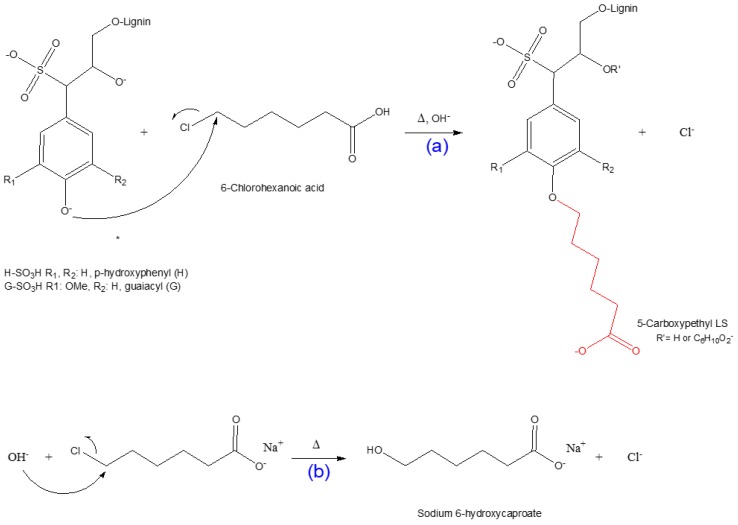
Scheme representation: (**a**) carboxypentylation reaction via the S_N_2 pathway; (**b**) production of sodium 6-hydroxycaproate (6-hydroxyhexanoic acid sodium salt as a side effect).

Since 6-chlorohexanoic acid is a primary halide, it can be predicted that the synthesis of the 5-carboxypentylated lignosulfonate proceeds through an S_N_2 nucleophilic substitution reaction [33,66,69]. The scheme of this reaction is depicted in Scheme 2. Under alkali conditions, NaOH reacts with the hydroxyl group of lignosulfonate and creates an alkoxide ion. This strong nucleophile approaches the alkyl halide from the back side of the C–Cl bond; as the C-nucleophile bond forms, the C–Cl bond breaks (Scheme 2a). At the transition state of the reaction, there is a 5-coordinate trigonal bipyramidal carbon for a femtosecond [66,69]. There is a possible competing side reaction, including the production of sodium 6-hydroxycaproate, which lowers the overall yield of the 5-CPELS as illustrated in Scheme 2b.

### 3.3. Mechanisms of Methylation of Lignosulfonate

**Scheme 3:**
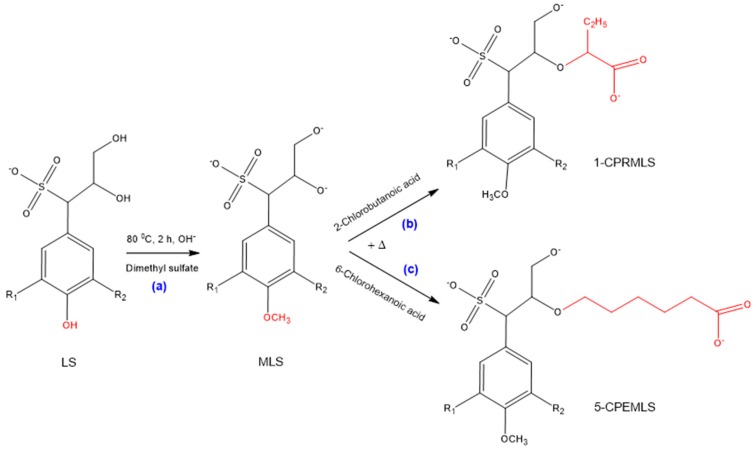
Possible reaction scheme for production of (**a**) methylated lignosulfonate (MLS); (**b**) 1-CPRMLS; (**c**) 5-CPEMLS.

The selectivity of the phenolic and aliphatic hydroxyl groups of lignosulfonate for the purpose of carboxypropylation and carboxypentylation can be evaluated by methylation processes [63,64]. Upon these reactions, firstly, LS was methylated, which protected all phenolic hydroxy groups of lignosulfonate without impacting the other parts of lignosulfonate structure [70], as shown in Scheme 3a, and then the methylated lignosulfonate (MLS) was reacted with 2-chlorobutanoic acid and 6-chlorohexanoic acid. The possible products of 1-CPRMLS and 5-CPEMLS, which may have been resulted from the substitution of hydroxy groups on the aliphatic chains of MLS with carboxyalkyl functions, are exhibited in Scheme 3b,c [20].

### 3.4. ^1^H-NMR Analysis

**Figure 1:**
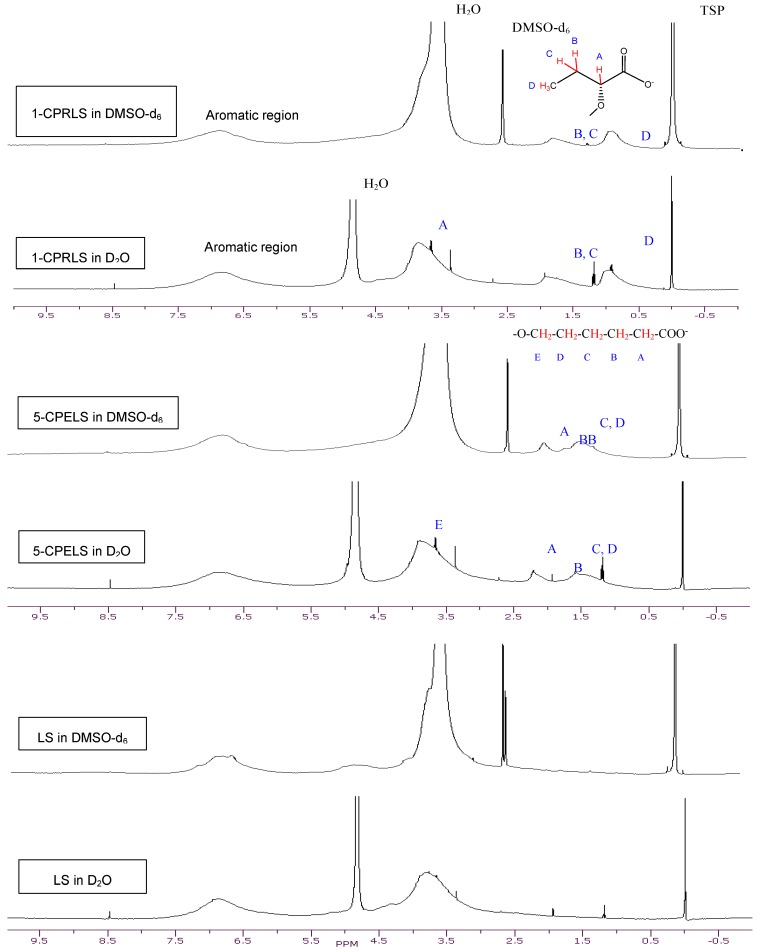
^1^H-NMR spectra of LS, 1-CPRLS, and 5-CPELS in DMSO-d_6_ and D_2_O solutions.

The ^1^H-NMR spectra of LS, 1-CPRLS, and 5-CPELS in D_2_O or DMSO-d_6_ were studied for the qualification of the structures of these lignin derivatives (Figure 1). In all spectra, protons in water appear at 4.7 (in D_2_O solutions) or 3.33 ppm (in DMSO-d_6_ solutions), and the sharp peak at 2.54 ppm corresponds to the protons of dimethyl sulfoxide [33]. In 1-CPRLS’ spectrum, the small broad peaks at δ ≈ (0.75–1.15) and (1.5–2.0) ppm can be principally assigned to the hydrogens of D and B-C (Figure 1), which is not present in LS’ spectrum [71]. In the proton spectrum of 5-CPELS, there are three small signals in the range of δ ≈ 1.25–2.35 ppm, which can be assigned to protons of C-D (1.25–1.65 ppm), B {1.60(+0.5)–1.90(+0.5) ppm}, and A {1.90(+0.5)–2.30(+0.5) ppm}, as they disappeared from LS’ spectrum (Figure 1) [72]. Furthermore, the hydrogens of A and E in 1-carboxypropyl and 5-carboxypentyl lignosulfonates appeared at 3.4–4.1 ppm, respectively.

**Figure 2:**
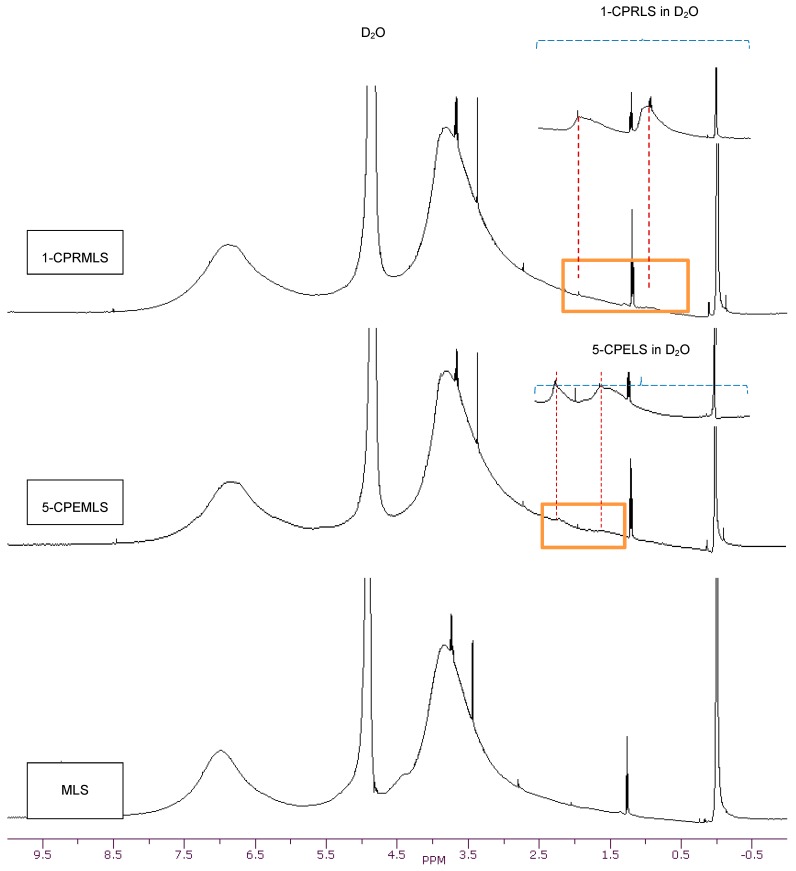
^1^H-NMR spectra of MLS, 1-CPRMLS, and 5-CPEMLS in D_2_O solutions at 25 °C.

Additionally, dimethyl sulfate (Scheme 3a) was used for synthesizing methylated lignosulfonate (MLS) [20,63,64]. The representative qualitative ^1^H-NMR spectrum supports the theory that dimethyl sulfate can afford the selective methylation of the phenolic hydroxyl groups in lignosulfonate [20]. Alternatively, the aliphatic hydroxyl groups in lignosulfonate remained unaffected [20]. In the carboxypropylation and carboxypentylation of MLS, the aliphatic hydroxyl groups may be substituted with carboxypropyl or carboxypentyl functions, generating 1-CPRMLS and 5-CPEMLS (Scheme 3b,c). ^1^H-NMR spectra of MLS, 1-CPRMLS, and 5-CPEMLS in D_2_O are shown in Figure 2. In contrast to that of 1-CPRLS, two weak peaks located at δ ≈ 0.75–1.15 and 1.5–2.0 ppm ranges are observable in 1-CPRMLS spectrum, and this demonstrates that the formation of 1-carboxypropyl MLS was ineffective. This limited efficiency can be attributed to two reasons: (1) the ionization efficiency of the aliphatic hydroxyl group is about 80 times lower than that of their phenolic counterparts [64]; and (2) steric hinderance may affect the reactivity of the nucleophile and the carbocation intermediate through the S_N_1 pathway in the production of 1-CPRMLS (Scheme 3b). Similarly, some broad and weak peaks in the range of δ ≈ 1.25–2.30 ppm were detected in the 5-CPEMLS spectrum. In this case, the reduction in the reactivity of chemical and thus limited sulfoproplyation can be related to the large gap between the halogen and the carboxyl group.

### 3.5. ^13^C-NMR Technique

**Figure 3:**
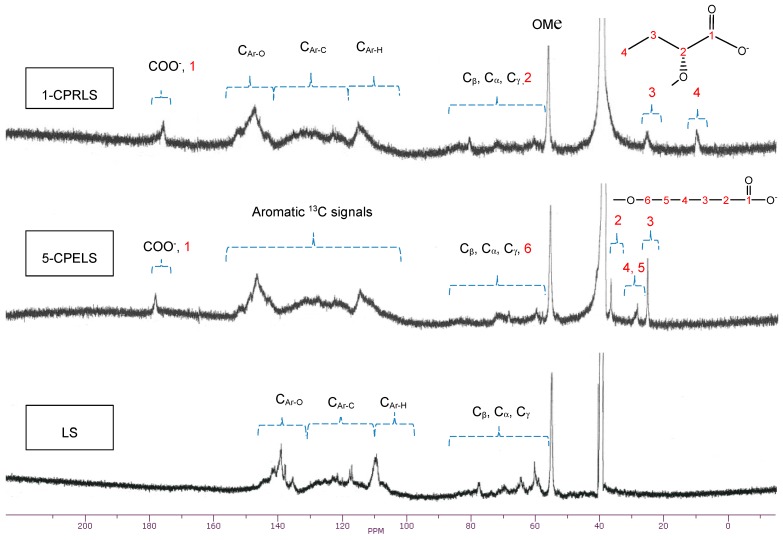
^13^C-NMR spectra of LS, 1-CPRLS, and 5-CPELS in DMSO-d_6_ solutions at 50 °C.

Generally, ^13^C-NMR analysis is one of the most reliable techniques for the comprehensive analysis of lignin derivatives [67,73]. Figure 3 shows the ^13^C-NMR spectra of LS, 1-CPRLS, and 5-CPELS. Table 1 also summarizes the chemical shift assignments of the structural features of LS, 1-CPRLS, and 5-CPELS products in the ^13^C-NMR analysis [62,67,68,74,75,76,77,78,79,80,81,82,83,84,85,86,87,88,89,90,91,92,93]. The signal at 56 ppm reflects the presence of a methoxy group [62]. The locations of the carboxylic acid peaks at 174–179 ppm [74,80,85], and methylene (−CH_2_−) and/or terminal methyl (-CH_3_) signals at 8–27 ppm [75,76,86,89,90] for 1-CPRELS and 22–38 ppm [75,86,90] for 5-CPERLS confirm the success of the carboxypropyl and carboxypentyl reactions for producing the 1-CPRLS and 5-CPELS, respectively. However, the mentioned signals did not exist in the ^13^C-NMR spectrum of LS (Figure 3).

### 3.6. FT-IR Spectrum Analysis

**Figure 4:**
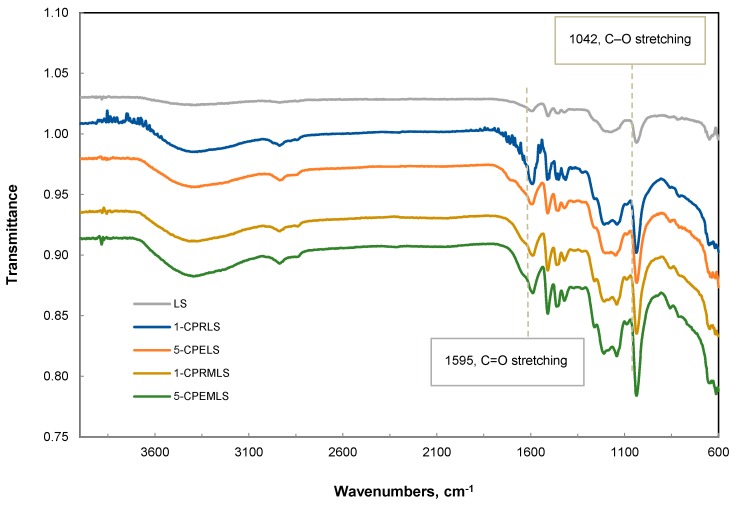
FT-IR spectra for LS, 1-CPRLS, 5-CPELS, 1-CPRMLS, and 5-CPEMLS.

To analyze the carbonyl groups of LS, 1-CPRLS, 5-CPELS, 1-CPRMLS, and 5-CPEMLS more accurately, FT-IR spectra of these samples were compared, as in Figure 4. Compared with the peak of LS, one noticeable difference in peak intensities at 1595 cm^−1^ appeared in the spectra of 1-CPRLS, 5-CPELS, 1-CPRMLS, and 5-CPEMLS, which is assigned to C = O stretching [20]. These results document the presence of the carboxypropyl and carboxypentyl side chains and successful formations of 1-CPRLS, 5-CPELS, 1-CPRMLS, and 5-CPEMLS. In addition, it can be seen that this peak for 1-CPRLS and 5-CPELS was stronger than that for 1-CPRMLS and 5-CPEMLS, which confirms the reduction of reactivity of aliphatic hydroxyl groups compared with phenolic groups in reacting with 2-chlorobutanoic acid and 6-chlorohexanoic acid in the carboxypropylation and carboxypentylation reactions (i.e., confirming the results of ^1^H-NMR analysis). Additionally, the peak corresponding to the C–O stretching of C–O–C was observed at 1042 cm^−1^ [95,96]. The results show an increase in the absorption of this peak for 1-CPRMLS and 5-CPEMLS compared to for 1-CPRLS and 5-CPELS. Based on this analysis, it is claimed that the -OH of the phenolic groups of LS was substituted with -OCH_3_ groups after methylation treatment.

### 3.7. Taguchi Method and Optimization

The Taguchi method is a systematic approach to finding the optimal combination of input parameters. A four-factor, three-level Taguchi orthogonal array (OA) design was used to study the effect of reaction parameters on the carboxypropylation and carboxypentylation of LS. The selected process variables were temperature (60, 70, and 80 °C), ClCBA_i_/LS (1.0, 1.5, and 2.0 mol·mol^−1^), reaction time (1.0, 1.5, and 2.0 h), and water/isopropyl (H_2_O/IPA) ratio (10%, 15%, and 20% *v*/*v*). ClCBA_i_ was used as an abbreviation for chloro carboxylic acid including ClCBA_1_ for 2-chlorobutanoic acid, and ClCBA_2_ for 6-chlorohexanoic acid in the synthesis of 1-CPRLS or 5-CPELS, respectively. The results of these experiments are reported in Table 2.

The dependent variables were the charge density (CD) and molecular weight (Mw). The optimal reaction conditions for the production of 1-CPRLS and 5-CPELS with the highest CD and Mw could be obtained by systematically analyzing the results of CD and Mw in Table 2. Interestingly, the optimal conditions for the carboxypropylation and carboxypentylation of lignosulfonate were NaOH concentration of 30 wt.%, temperature of 80 °C, time of 2 h, ClCBA_i_/LS of 1.0 mol·mol^−1^, and H_2_O/IPA of 15 vol.%. The 1-CPRLS and 5-CPELS prepared under these conditions showed the highest CDs of −3.45 and −2.94 meq g^−1^ and molecular weights of 87,900 and 42,400 g mol^−1^, respectively.

### 3.8. Effect of Reaction Conditions on DS

**Figure 5:**
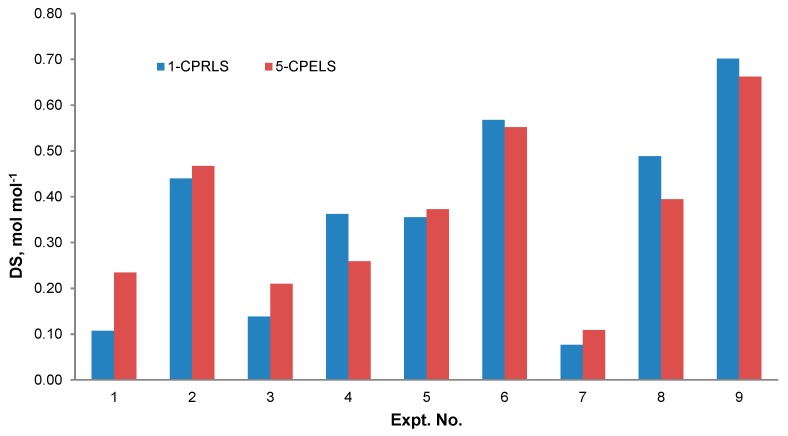
Degree of substitution (DS) for different samples produced under various reaction conditions stated in Table 2 for the production of 1-CPRLS and 5-CPELS.

Figure 5 shows the relationship between the reaction conditions and the DS of 1-CPRLS and 5-CPELS. As can be seen, the DS significantly depended on the reaction conditions. Maximum DSs of 0.70 and 0.66 mol·mol^−1^ were obtained under the conditions of 1.0 mol mol^−1^ of ClCBA_i_/LS ratio, 80 °C, 2 h, 15 vol.% of H_2_O/IPA ratio, and 30 wt.% NaOH for 1-CPRLS and 5-CPELS, respectively. As the reactions were endothermic, the increase in temperature facilitated the reaction and thus increased the DS [97]. Additionally, the highest DS was found for the 15 % H_2_O/IPA ratio of the reaction medium. Water plays a significant role in the carboxypropylation and carboxypentylation of LS by facilitating the diffusion of reagents and making them more accessible to lignosulfonate [98,99]. However, the decrease in the DS at the water content of 20% could be due to a dilution effect. It has been reported that a mixture of isopropanol, water, and NaOH forms a two-phase liquid system consisting of IPA, water, and a small quantity of NaOH in the solvent-rich layer; and sodium hydroxide, water, and a very small amount of IPA in the water-rich one [97,100]. In this case, increasing the water proportion to 20 vol.% in the reaction medium would lower the concentrations of both the reagent and the nucleophile in the organic phase, hampering the reaction rate [97]. Furthermore, the maximum DS was obtained for the samples reacted for 2 h. Thus, prolonging the reaction time provided favourable conditions for carboxyalkylation (i.e., better contact between the etherifying agent and lignosulfonate) [31,98]. Finally, the best ratio of ClCBA_i_/LS in this work was 1.0 mol.mol^−1^ for both reactions. With further increase of this ratio from 1.0 to 2.0 mol·mol^−1^, the undesired products were probably produced more dominantly, and the DSs of 1-CPRLS and 5-CPELS decreased [31].

### 3.9. Properties of LS, MLS, 1-CPRLS, 5-CPELS, 1-CPRMLS, and 5-CPEMLS

Table 3 summarizes the properties of LS, MLS, 1-CPRLS, 5-CPELS (produced under the optimal conditions), 1-CPRMLS, 5-CPEMLS, as well as 1-CELS reported in the literature [20]. As can be seen, there was a higher carboxylic acid group content for 1-CPRLS and 5-CPELS compared with LS (2.37 and 2.13 vs. 0.08 mmol·g^−1^, respectively), which reflects the success of the carboxyalkylation of LS. The higher carboxylate group content of 1-CPRLS compared to 5-CPELS may be due to the lower reactivity of 6-chlorohexanoic acid compared to 2-chlorobutanoic acid, as stated earlier. The longer chain hydrocarbon of 2-chlorobutanoic acid than in 2-chloropropionic acid (i.e., the carboxyl donor in 1-CELS production) is a possible reason for the lower carboxylic acid group content of 1-CPRLS compared to 1-CELS [20]. Furthermore, 1-CPRLS with a higher molecular weight (87,900 g·mol^−1^ for 1-CPRLS compared with 42,400 and 46,500 g mol^−1^ for 5-CPELS and 1-CELS) may equip the polymer with a coagulating affinity for solution systems (e.g., dye solutions).

As stated earlier, methylated lignosulfonate (MLS) was synthesized for covering the phenolic hydroxide group of LS (Scheme 3a) [20]. On one hand, the phenolic group contents determined for MLS, 1-CPRMLS, and 5-CPELMS were 0.09, 0.07, and 0.08 mmol·g^−1^, respectively, which along with the results of Figure 2, confirmed the success of methylation reaction. On the other hand, their carboxylic acid group contents (0.03, 0.51, and 0.37 mmol·g^−1^ for MLS, 1-CPRMLS, and 5-CPELMS, respectively) indicate that the aliphatic hydroxy groups of MLS, and consequently LS, can be substituted with carboxypropyl and carboxypentyl groups in the carboxypropylation and carboxypentylation reactions. In addition, the Mws of MLS, 1-CPRMLS, and 5-CPELMS reported in Table 3 were 40,100, 34,400, 27,000 g·mol^−1^, respectively. This sequence of declining molecular weight (5-CPELMS < 1-CPRMLS < MLS) could be due to an alkaline hydrolysis occurring during the grafting reactions for producing lignin derivatives [20]. Evidently, 5-CPELMS and 1-CPRMLS experienced an extra hydrolysis during the two-step reactions of methylation and carboxyalkylation [20].

The empirical formula of LS, MLS, 1-CPRLS, 5-CPELS, 1-CPRMLS, and 5-CPEMLS are also given in Table 3. Elemental analysis included the C_9_ formula of C_9.00_H_9.47_S_0.45_O_5.71_, C_9.00_H_10.87_S_0.35_O_6.53_, and C_9.00_H_10.98_S_0.34_O_6.01_ for LS, 1-CPRLS, and 5-CPELS, respectively. It is evident that the oxygen and hydrogen contents were higher for 1-CPRLS and 5-CPELS than for LS, confirming the success of carboxypropylation and carboxypentylation [31]. The yield of the products was also in a similar order, even though a slightly higher yield was obtained for 1-CELS (Table 3).

### 3.10. Dye Removal

**Figure 6:**
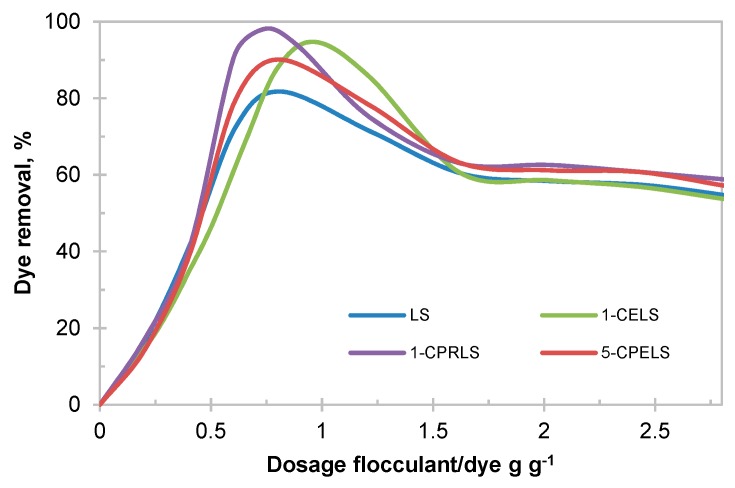
Impacts of dosage of LS, 1-CELS, 1-CPRLS, and 5-CPELS on the removal of ethyl violet (EV) dye from solution.

In this work, a 100 mg·L^−1^ dye concentration was chosen as a model wastewater, which is based on the available literature (i.e., 50–250 mg L^−1^ dye concentration in the wastewater of the textile industry) [101]. Figure 6 depicts the impact of lignosulfonate derivatives on the removal efficiency of EV cationic dye from the model wastewater samples. As is evident, the maximum EV removal was found between 82 and 98 wt.% for LS, 1-CELS, 1-CPRLS, and 5-CPELS (Figure 6). The results confirm the improved performance of modified lignosulfonate samples—particularly 1-CPRLS—in removing the EV dye.

According to Table 3, lignosulfonate derivatives had a higher charge density and molecular weight than LS (except for methylated samples), and these properties have profound impacts on their interaction with dye molecules in solutions [28,29,102,103]. In this study, the presence of carboxylate groups along with different carboxyalkylated chains (1-CELS, 1-CPRLS, and 5-CPELS) provided a large number of available adsorption/interaction sites for dyes [104]. The removal mechanism could be explained by the combination of charge neutralization and polymer bridging, which led to their agglomeration and thus removal from the solution. The higher efficiency for 1-carboxypropyl lignosulfonate at the optimum dosage (0.75 g·g^−1^) of 1-CPRLS/EV was due to its higher charge density and molecular weight (Table 3). The reduction in dosage needed for the removal and increased removal efficiency are critical factors in the dye removal, as a smaller dosage would generate a smaller amount of precipitated wastes. A combination of higher removal and lower dosage would also lead to precipitated flocs with a higher proportion of dyes. This lesser use of lignin polymer would help to reduce the cost of this flocculation process. It was also seen that the optimized dosages for lignosulfonate derivatives were in a smaller range than that for lignosulfonate, which stems from the higher charge density of the lignosulfonate derivatives. At a higher charge density, a small dosage increase may lead to the repulsion of dye segment or dye/lignosulfonate flocs with anionic charges that may repel each other, leading to more limited dye removal [102]. Compared with 1-CELS, 1-CPRLS was more effective at a lower required dosage, which is attributed to the higher molecular weight of 1-CPRLS. 

### 3.11. Carboxyalkylated Lignosulfonate Comparison

The results of this work confirmed that changing the type of carboxylic acid group donor will have a considerable impact on the mechanism of the carboxyalkylation reaction of lignosulfonate, the reaction conditions required for achieving the best results, and hence on the properties of carboxyalkylated lignosulfonate. In addition, due to the differences in charge density and molecular weights of these modified lignosulfonates, they would have different flocculation performance and dye removal efficiency if used as coagulants (Figure 6). In this work, we introduced a more appealing reaction system using less solvent (i.e., 30 vol.% in this work vs. more than 90 vol.% in our previous work) [20] for producing a carboxyalkylated lignosulfonate with better properties. The use of less solvent for modifying lignosulfonate, while generating a better lignosulfonate derivative, will have less significant environment footprints and be more accommodative for the chemical industry.

## 4. Conclusions

In this work, 1-carboxypropyl and 5-carboxypentyl lignosulfonates were successfully produced under different conditions, and the mechanism of the grafting reactions and the properties of the products were comprehensively analyzed by FT-IR, ^1^H-NMR, and ^13^C-NMR analyses. The methylation experiment confirmed the limited attachment of the carboxyethylate group to the aliphatic hydroxy groups of lignosulfonate. The impacts of the reaction conditions using Taguchi orthogonal design on the charge density, molecular weight, and degree of substitution for 1-CPRLS and 5-CPELS were also studied. The maximum CDs of −3.45 and −2.94 meq g^−1^, Mws of 87,938 and 42,364 g·mol^−1^, and DSs of 0.70 and 0.66 mol·mol^−1^ were obtained for 1-CPRLS and 5-CPELS, respectively, under the optimum conditions of 1.0 mol·mol^−1^ of 2-chlorobutanoic acid or 6-chlorohexanoic acid/lignosulfonate ratio, 80 °C, 2 h, and 15% *v*/*v* of H_2_O/IPA. The 1-CPRLS and 5-CPELS (produced under the optimum conditions), as well as 1-CELS, were applied for removing EV dyes, and the results confirmed the superior efficiency of 1-CPRLS as a coagulant for the dye removal due to its higher charge density and M_W_.

## Figures and Tables

**Table 1:** Structural assignments in ^13^C-NMR spectra of LS, 1-CPRLS, and 5-CPELS in DMSO-d_6_ solutions based on the spectra in Figure 3.

Selected Groups (ppm)	δ_Exp._ (ppm)			Peak Assignment According to Literature Data	δ (ppm)
	LS	1−CPRLS	5−CPELS		
Carbonyl C (C = O) (200.0–160.0) [85]	-	178−174	179−177	Carboxylic acid units	173.0 [86], (172.8–171.2, 170.7–168.8) [76], 172.8 [81]
Aromatic C–O (ArC_3,_ C_4_) (160.3–144.0) [75] * (160.0–140.0) [76] (154.6–140.7) [93]	148–136	160–141	156–140	ArC_4_ in H-type units	157.8 [75] *, 156.4 [67,78]
				ArC_3_/C_3′_ in etherified 5-5 units	152.1 [67]
				ArC_4_ in etherified G-type units with αC = O	151.3 [75] *
				ArC_3_ in etherified G-type β-O-4 units	(151.5–151.1) [75] *, 149.1 [67]
				ArC_3_ in etherified G-type units	149.7 [89], 149.4 [67,77], 149.0 [75] *
				ArC_3_/C_3′_ in pinoresinol structure	148.6 [74]
				ArC_4_ in etherified G-type units	148.2 [75] *, 146.8 [67,77,89]
				ArC_3_ in non-etherified G-type β-O-4 units	(148.7–148.1) [75] *, 146.6 [67]
				ArC_4_ in non-etherified G-type β-O-4 units	(147.1–146.6) [75] *, 145.8 [67,77,89]
				ArC_4_/C_4′_ in pinoresinol structure	146.7 [74]
				ArC_4_/C_4′_ in etherified 5-5 units	145.0 [74]
				ArC_4_/C_4′_ in non-etherified 5-5 units	144.0 [75] *,143.3 [67,89]
				ArC_4_ in ring β of β-5 units	143.3 [67,89]
Aromatic C–C (ArC_2,_C_5_ condensed) (140.7–123.4) [93] (140.3–122.9) [93] * (140.0–123.0) [76]	136–114	141–118	140–118	ArC_1_ in etherified G-type units	137.5 [75] *, 134.6 [67,77]
				ArC_1_/C_1′_ in pinoresinol structure	132.9 [74]
				ArC_5_/C_5′_ in etherified 5-5 units	132.4 [67,77,89]
				ArC_1_ in non-etherified 5-5 units	131.2 [75] *, 131.1 [67,89]
				ArC_2_/C_6′_ in H-type units	128.6 [92], 128.1 [67], 128.0 [86]
				ArC_5_/C_5′_ in non-etherified 5-5 units	126.8 [75] *, 125.9 [67,77,89]
Aromatic C–H (ArC_2,_C_5,_C_6_–H) (131.5–108.5) [50] * (123.4–105.3) [67] (123.0–103.0) [51]	113–106	118–107	118–100	ArC_6_ in G-type units	(125.2–120.0) [75] *, 124.0 [75], (123.0–117.0) [76], (119.9, 119.4, 118.4) [67,89], 118.4 [77]
				ArC_5_ in G-type units	(118.1–111.4) [75] *, (117.0–114.0) [76], (115.1, 114.7) [67,77,89]
				ArC_5_/C_5′_ in pinoresinol structure	115.7 [74]
				ArC_3_,C_5_ in H-type units	115.0 [75]
				ArC_2_ in G-type units	(115.2–111.3) [75] *, (114.0–108.0) [76], (111.1–110.4) [67,89]
				ArC_2_/C_2′_ in pinoresinol structure	110.3 [74]
C_α_,C_β_, C_γ_ (90.0–58.6) [93] (89.8–58.6) [93] (81.9–56.3) [75] *	80–58	86–59	89–60	C_β_ in β-O-4, C_α_ in β-5 and β-β units	(90.0–82.5) [88], (90.0–78.0) [76]
				C_α_ in pinoresinol structure	87.0 [74]
				C_α_ in β-5 and G-type β-5 units	86.7 [92], 86.6 [67,89], (68.2–68.7) [75] *
				C_β_ in G-type β-O-4 units	(84.6–83.8) [67],84.2 [92], (81.9–77.7) [75] *
				C_α_, C_γ_ in β-β units	(84.3, 71.1) [92]
				C_α_ in β-O-4 and G-type β-O-4 units	(78.0–67.0) [76], 72.8 [92], (71.8, 71.2) [67,89], 71.2 [77], (68.0, 66.7) [75] *
				C_γ_ in β-β units	72.4 [89]
				C_γ_ in pinoresinol structure	72.3 [74]
				C_γ_ in β-O-4 and G-type β-O-4 units with αC = O	63.2 [67,89], 53.7 [75] *
				C_γ_ in G-type β-5 and β-1 units	62.8 [67,77,89]
				C_γ_ in β-O-4 and G-type β-O-4 units (without αC = O)	(62.8, 62.7) [75] *, (61.3–58.0) [76], 60.2 [67,77,89], (59.0–61.0) [88]
Methoxyl, C_β_ in β-β and β-5 units (52.0–58.0) [76]	58–52	59–54	59–53	Methoxyl (-OCH_3_)	(58.0–54.0) [76], 56.5 [92], 56.2 [74], 56.0 [62,86,87], 55.6 [67,89]
				Methoxyl and C_β_ in β-5 units	(58.1–52.5) [93], (56.5–52.0) [75] *
				C_β_ in pinoresinol structure	54.8 [74]
				C_β_ in β-β and β-5 units	(54.0–53.0) [88], (54.0–52.0) [76]
				C_β_ in β-β units	53.8 [77], 53.9 [67,89]
				C_β_ in β-5 units	53.4 [67,77,89]
Alkyl C (10.0–45.0) [90] (0.0–45.0) [86]	-	27–8	38–22	Terminal methyl (-CH_3_) and methylene (-CH_2-_) in saturated aliphatic chain	45–10 [90], 41.2–12.1 [75] *, 26.7 [89]
				Methylene groups in aliphatic rings and side chains	45–25 [86], 29.2 [89], 27 [76]
				Terminal methyl groups	25–0 [86], 12–15 [76], 14.0 [89]

* Lignosulfonate assignments in MeOH-d_4_. The ^13^C solvent shifts Δδ_1_ = δ(DMSO)−δ(MeOH-d_4_) varies from −3.25 to +4.06 ppm [94]. The change in chemical shift of the sulfonated compounds compared to the corresponding compound with a hydroxyl group was reported in MeOH-d_4_. Significant chemical shift differences, Δδ_2,_ are observed for the 1, 2, 6, α and β-positions, of approximately +7, −3.5, −4, +(6–8) and +5 ppm for ^13^C NMR. Smaller changes are observable for γ-position, whereas the other positions only show minor changes [75]. Carbon elements in dimethyl sulfoxide appear at 39.5 [88] (or 39.53 ± 05) ppm [88].

**Table 2:** Levels and dependent variables for the control factors in the Taguchi orthogonal array (OA) design (L_9_).

Expt. No.	Control Factors and Their Levels				Dependent Variables			
					1-CPRLS		5-CPELS	
	Temp. (°C)	ClCBA_i_ */LS (mol·mol^−1^)	Time (h)	H_2_O/IPA (vol.%)	CD (meq·g^−1^)	Mw (g·mol^−1^)	CD (meq·g^−1^)	Mw (g·mol^−1^)
1	60	1.0	1.0	10	3.04	33,159	2.69	34,124
2	70	2.0	1.0	15	3.25	56,234	2.81	36,413
3	80	1.5	1.0	20	2.47	48,437	2.82	24,302
4	60	1.5	1.5	15	3.14	52,455	2.75	39,009
5	70	1.0	1.5	20	3.30	32,490	2.93	32,070
6	80	2.0	1.5	10	3.37	81,175	3.12	38,681
7	60	2.0	2.0	20	3.17	30,496	2.86	33,787
8	70	1.5	2.0	10	3.35	61,665	2.08	41,958
9	80	1.0	2.0	15	3.45	87,938	2.94	42,364

* ClCBA_i_ = Chloro carboxylic acid including ClCBA_1_ (2-chlorobutanoic acid for 1-CPRLS production) and ClCBA_2_ (6-chlorohexanoic acid for 5-CPELS production). In all reactions, NaOH was 30 wt.%.

**Table 3:** Properties of LS, 1-CPRLS, 5-CPELS, MLS, 1-CPRMLS, and 5-CPEMLS.

Sample	CD (meq·g^−1^) (±0.10)	Carboxylic acid Group Content (mmol·g^−1^) (±0.10)	DS (mol·mol^−1^) (±0.02)	Phenolic-OH Group Content (mmol·g^−1^) (±0.10)	Empirical Formula (* the basic unit)	Mw (g·mol^−1^)	Mn (g·mol^−1^)	Mw/Mn	Yield (%) (±0.1)
LS	2.21	0.08	0.02	0.81	C_9.00_H_9.47_ S_0.45_O_5.71_	32,506	19,641	1.66	-
1-CPRLS	3.45	2.37	0.70	0.43	C_9.00_H_10.87_S_0.35_O_6.53_	87,938	18,044	4.87	83.4
5-CPELS	2.94	2.13	0.66	0.69	C_9.00_H_10.98_S_0.34_O_6.01_	42,364	14,961	2.83	80.5
MLS	2.17	0.03	0.01	0.09	C_9.00_H_9.82_S_0.44_O_5.46_	40,086	16,145	2.48	-
1-CPRMLS	2.29	0.51	0.12	0.07	C_9.00_H_10.40_S_0.25_O_5.78_	34,417	9152	3.76	-
5-CPEMLS	2.25	0.37	0.09	0.08	C_9.00_H_9.18_S_0.27_O_5.63_	27,014	5908	4.57	-
1-CELS	3.51 [20]	2.63 [20]	0.78 [20]	0.34 [20]	C_9.00_H_9.78_ N_0.05_S_0.28_O_6.20_ [20]	46,493 [20]	26,209 [20]	1.77 [20]	87.8

* For a C_9_ unit of lignosulfonate.
